# Photoprotection in pregnancy: addressing safety concerns and optimizing skin health

**DOI:** 10.3389/fmed.2025.1563369

**Published:** 2025-03-27

**Authors:** Henry W. Lim, Jaime Piquero-Casals, Sergio Schalka, Giovanni Leone, Carles Trullàs, Anthony Brown, Monica Foyaca, Yolanda Gilaberte, Jean Krutmann, Thierry Passeron

**Affiliations:** ^1^Division of Photobiology and Photomedicine, Department of Dermatology, Henry Ford Health, Detroit, MI, United States; ^2^Department of Dermatology, Dermik Multidisciplinary Dermatological Clinic, Barcelona, Spain; ^3^Medcin Skin Research Center and Biochemistry Department, Chemistry Institute of São Paulo University, São Paulo, Brazil; ^4^Photodermatology and Vitiligo Treatment Unit, Israelite Hospital, Rome, Italy; ^5^Innovation and Development, ISDIN, Barcelona, Spain; ^6^Department of Dermatology, Miguel Servet University Hospital, IIS Aragón, Zaragoza, Spain; ^7^IUF - Leibniz-Institut für umweltmedizinische Forschung, Düsseldorf, Germany; ^8^Department of Dermatology, Centre Hospitalier Universitaire de Nice, Université Côte d’Azur, Nice, France; ^9^Centre Méditerranéen de Médecine Moléculaire, INSERM U1065, Université Côte d’Azur, Nice, France

**Keywords:** pregnancy, breastfeeding, ultraviolet radiation, visible light, photoprotection, sunscreens, endocrine-disrupting chemicals, lactation

## Abstract

Pregnancy is associated with physiological skin changes, altered response to UV exposure and increased risk of pigmentary disorders such as melasma and linea nigra, which can impact quality of life. This review explores the effects of photoprotection during pregnancy, focusing on safety, efficacy, and the role of sunscreens in preventing pregnancy-associated hyperpigmentation and UV-induced skin damage. Sunscreen use in pregnant women is generally low, despite evidence supporting the benefits of broad-spectrum sunscreens to mitigate pigmentation changes and prevent DNA damage from UV exposure. Physiological changes during pregnancy influence sunscreen selection; ideally, sunscreens should be mineral-based, cosmetically acceptable, potentially supplemented with safe organic filters to optimize cosmetic acceptability and adherence, and free from ingredients associated with potential risks during pregnancy. Tinted sunscreens, which provide protection against high-energy visible light (HEVL), may offer enhanced prevention of hyperpigmentary disorders, and are recommended due to their added camouflage benefits, though shade options should ideally match diverse skin tones. Photoprotection strategy should include the use of wide-brimmed hats, sun-safe clothing and regular use of high-SPF, broad-spectrum sunscreens that protect against UVB, UVA, and HEVL. Tinted, mineral-based formulations potentially supplemented with safe organic filters may be optimal for pregnant women providing both effective protection and cosmetic benefits.

## 1 Introduction

Pregnancy and lactation represent unique physiological states in a woman’s life, characterized by profound hormonal, metabolic, and immunologic changes that can affect the skin (1). During this period, it is essential to carefully manage environmental exposures to ensure both maternal and fetal safety. Modifiable elements of the environment—such as diet, medications, and topical products—play a crucial role, as these can directly influence maternal and neonatal health outcomes. Photoprotection, in particular, becomes critical due to increased skin sensitivity and the higher likelihood of developing or exacerbating dermatologic conditions like autoimmune disorders, hyperpigmentation or melasma (2, 3).

Pregnant women may experience heightened susceptibility to UV-induced skin changes, making photoprotection a cornerstone of dermatologic care. Recommendations typically include physical barriers, such as wide-brimmed hats, sun-safe clothing, and parasols, along with broad-spectrum, SPF30 or above sunscreens, ideally specifically formulated for sensitive skin. These sunscreens should be carefully selected to minimize exposure to potentially harmful chemicals, particularly during the first trimester and the breastfeeding period, when the fetus or infant is most vulnerable.

This review provides an in-depth examination of photoprotection strategies during pregnancy and lactation, drawing on current evidence and expert insights. We evaluate sunscreen formulations that are deemed safe for this population and explore the impact of photoprotection on common pregnancy-associated skin conditions. Our goal is to provide obstetrician-gynecologists, dermatologists and healthcare providers with evidence-based recommendations for optimizing skin health and safety in pregnant and breastfeeding women.

## 2 Methods

We conducted a narrative review of the literature. We performed literature searches with PubMed from January 1990 to December 2021 using the keywords “melasma,” “pathogenesis,” “breastfeeding” “ultraviolet radiation,” “visible light,” “photoprotection,” and “sunscreen,” “endocrine-disrupting chemicals,” “lactation,” “UV radiation,” “skin changes,” and “pigmentary disorders.” The search was limited to English, Spanish and French language articles. Articles were selected depending on their relevance. We used a generative AI tool (ChatGPT, OpenAI, version January 2025) to assist with language refinement and improve manuscript readability.

## 3 Results and discussion

### 3.1 Skin changes during pregnancy and the role of photoprotection

Pregnancy brings about distinct physiological changes that enhance skin sensitivity and modify the body’s response to ultraviolet (UV) and visible light (VL) radiation (4). Three main features of pregnancy-associated skin changes highlight the critical need for photoprotection: (1) increased hormone levels heighten melanin production and distribution in the skin, particularly in the facial area; (2) pregnancy-associated hyperpigmentation, such as linea nigra and melasma, is often exacerbated by sun exposure; and (3) oxidative stress from UV and VL exposure is amplified in pregnant skin, making it more susceptible to damage and pigmentation changes (5). Pregnancy-related hyperpigmentation, commonly seen in dark skinned women, is more likely in individuals with high outdoor activity levels, with additional weekly outdoor exposure increasing the risk of pigmentation.

UV radiation (UVB, UVA) and VL (400–700 nm), especially high-energy visible light (HEVL) from 400 to 500 nm, play significant roles in pregnancy-associated pigmentation changes. HEVL and UVA1 can penetrate deeper into the skin, generating reactive oxygen species (ROS) and activating pathways that exacerbate pigmentation (6, 7). HEVL, in particular, stimulates tyrosinase activity in melanocytes via the photoreceptor Opsin 3, driving melanogenesis (8). This mechanism is particularly prominent in darker skin phototypes (IV-VI), where HEVL exposure induces persistent hyperpigmentation, while phototypes I-II are more susceptible to erythema following exposure (9, 10). However, recent studies suggest a more complex pigmentation response across different skin tones, showing that blue (450 nm) and green (530 nm) light can also stimulate melanogenesis in healthy human skin histocultures of fair-skinned individuals (SPT-I to SPT-III), in some cases even more effectively than UV radiation (11). Additionally, although blue light is the primary contributor to VL-induced pigmentation, green light also plays a role, albeit to a lesser extent (12). These findings challenge conventional assumptions and highlight the need for broad-spectrum photoprotection that addresses diverse pigmentation responses.

The heightened sensitivity to longer-wavelength solar radiations during pregnancy necessitates proactive and customized photoprotection. Broad-spectrum sunscreens with not only a high sun protection factor (SPF) but providing also an effective protection against UVA1 and HEVL are recommended (3). Tinted sunscreens, which provide additional protection through iron oxide pigments (and in some, pigmentary titanium dioxide), can be particularly beneficial, as they not only protect from HEVL but also offer cosmetic camouflage, catering to a range of skin tones (13).

### 3.2 Sunscreens in pregnant women and populations at risk

Certain groups of pregnant women are at a higher risk of developing or worsening sun-induced dermatoses, such as melasma, and therefore benefit particularly from consistent sun protection (14). Studies have demonstrated the effectiveness of regular sun protection for preventing and managing melasma during pregnancy (15). Women with a history of melasma or hyperpigmentation are highly susceptible to pigmentation changes during pregnancy, given the heightened skin sensitivity to ultraviolet (UV) and visible light induced by hormonal shifts (16). Studies indicate that women with darker skin tones (skin phototypes IV to VI) are especially vulnerable, as they are more likely to experience persistent hyperpigmentation triggered by visible light (17).

Sun exposure has broader health implications, particularly during pregnancy. Folic acid, essential for healthy pregnancy and the prevention of neural tube defects, is photodegraded by UV radiation. Studies have shown that UV exposure can reduce the effectiveness of folic acid supplementation in women of childbearing age (18). On the other hand, vitamin D, synthesized in the skin upon exposure to UVB radiation, is vital during pregnancy and lactation for the development of the skeletal system in early childhood (19).

Balancing vitamin D levels while avoiding excessive sun exposure remains a complex issue, especially during pregnancy. Although extensive literature review shows suggest that minimal sun exposure from daily activities, like brief outdoor errands, can suffice for achieving adequate vitamin D levels (20), other studies indicate that pregnancy may elevate vitamin D needs, potentially requiring more intentional sunlight exposure or vitamin D supplementation is this population (21).

Women living in high UV index areas, including tropical or subtropical regions, also face an increased risk of sun-induced skin changes. Given the intense sun exposure in these climates, daily use of broad-spectrum sunscreens, along with physical barriers like hats and protective clothing, is strongly recommended. Additionally, taking photosensitive drugs, or suffering from photosensitive or hyperpigmentary dermatoses making rigorous photoprotection an essential component of skincare during pregnancy. A study on 50 pregnant women found significant changes in skin permeability and pH, with sun exposure increasing permeability early on and notable shifts in pH and water loss around 28–30 weeks. These fluctuations in skin barrier function persisted across different skin types, seasons, and exposure levels (22).

By identifying and prioritizing sun protection for these high-risk groups, primary care physicians, obstetricians and dermatologists can help in preventing the onset or worsening of melasma and related dermatoses, supporting skin health throughout pregnancy.

#### 3.2.1 Pregnancy dermatoses and the role of sunscreens

Various pregnancy-related dermatoses, including pemphigoid gestationis, polymorphic eruption of pregnancy, intrahepatic cholestasis and atopic eruption of pregnancy, may not be directly influenced by sun exposure. However, photoprotection remains a crucial part of managing these conditions, as heat from sun exposure can exacerbate itching, and unprotected skin may develop post-inflammatory hyperpigmentation (23), although NB-UVB has been proposed as therapy to manage the pruritus of some of these dermatoses (24). Given that excessive ultraviolet radiation can contribute to both carcinogenesis and premature aging, healthcare professionals should emphasize photoprotective measures for pregnant or lactating women as an integral aspect of care (9).

On the other hand, pregnancy often triggers or exacerbates a range of facial dermatoses, with melasma, acne, rosacea, and certain autoimmune skin conditions frequently observed or worsened during this period (25–27). Hormonal changes, particularly elevated estrogen and progesterone, play a significant role in increasing skin sensitivity and reactivity, leading to hyperpigmentation and inflammatory lesions that can persist postpartum. Melasma is especially common, with exposure to sunlight a primary trigger for its onset and worsening (16). Similarly, hormonal fluctuations may lead to flare-ups in acne and rosacea, both of which can be further aggravated by UV exposure and environmental stressors (27, 28).

**FIGURE 1:**
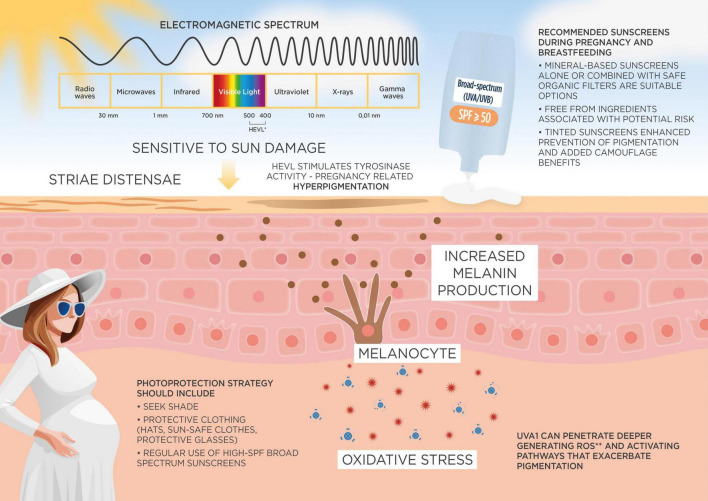
Skin changes during pregnancy and photoprotection strategies. This figure illustrates the physiological skin changes during pregnancy caused by altered response to UV exposure (UV) and visible light radiation, including heightened melanin production and the risk of hyperpigmentation such as melasma. It highlights the role of photoprotection strategies, including the use of broad-spectrum sunscreens with high SPF, wide-brimmed hats, and protective clothing. The recommended characteristics of sunscreens during pregnancy are summarized, focusing on mineral-based filters (e.g., zinc oxide, titanium dioxide), safety considerations, and cosmetic acceptability for improved adherence. *HEVL, High-Energy Visible Light (400–500 nm) and also green light (500–600 nm) participate (but in a much lesser extent compared to HEVL) to VL-induced pigmentation. **ROS, reactive oxygen species.

Sunscreens are an essential component in the prevention and management of these pregnancy-related dermatoses. Broad-spectrum sunscreens with UVA and UVB protection help protect the skin from further irritation and hyperpigmentation, especially critical for melasma patients, as UV exposure can significantly darken existing pigmentation. Mineral sunscreens, often recommended for pregnant women due to their reduced risk of absorption and minimal irritant properties, provide a protective layer on the skin’s surface, limiting the impact of UV (Figure 1). Regular sunscreen use can thus mitigate the appearance of melasma and prevent flare-ups of acne and rosacea, aiding in the maintenance of a more stable skin condition during pregnancy (23).

As part of a comprehensive skincare regimen, photoprotection is also beneficial in managing autoimmune-related dermatoses, which can sometimes worsen with sun exposure. By reducing inflammation and avoiding triggers that exacerbate skin vulnerability, sunscreens support overall skin health during pregnancy (29). Primary care physicians, obstetrician-gynecologists and dermatologists play a key role in advising pregnant patients on suitable sunscreen products, ideally those with a high safety profile and minimal endocrine-disrupting ingredients, to help prevent and control these dermatoses effectively and safely throughout gestation (30, 31).

#### 3.2.2 Safe UV filters for pregnant and breastfeeding women: key considerations

When selecting sunscreens for pregnant and breastfeeding women, mineral filters like titanium dioxide and zinc oxide are often recommended as the first choice due to their safety profiles and minimal systemic absorption after topical application (32). These inorganic filters provide broad-spectrum protection with a low concern about endocrine disruption or bioaccumulation, making them a reliable option for this population. However, the primary limitation of mineral filters lies in their cosmetic acceptability, as they may leave a white cast or feel heavier on the skin.

To address these challenges, combining inorganic filters with specific organic filters can improve the cosmetic properties of sunscreens making them more appealing for daily use while maintaining safety and efficacy (Table 1). Organic filters such as ethylhexyl triazone, bis-ethylhexyloxyphenol methoxyphenyl triazine, butyl methoxydibenzoylmethane, phenylbenzimidazole sulfonic acid, diethylamino hydroxybenzoyl hexyl benzoate and organic particulate filters such as methylene bis-benzotriazolyl-tetramethylbutylphenol and tris-biphenyl triazine provide UV protection with low systemic absorption.

**TABLE 1:** Characteristics of recommended sunscreens during pregnancy and breastfeeding.

Characteristic	Recommendation
Type of filters	Mineral-based (zinc oxide, titanium dioxide) preferred; safe organic filters for improved sensorial profile and better adherence.
Broad-spectrum protection	High SPF with UVA, UVB, and HEVL* coverage.
Endocrine disruption	Avoid filters with potential endocrine-disrupting properties (e.g., oxybenzone, octinoxate, 4-methylbenzylidene camphor).
Cosmetic acceptability	Lightweight, non-sticky texture, minimal white cast, tinted formulations to match diverse skin tones.
Additional ingredients	Safe antioxidants (e.g., vitamin E, niacinamide), moisturizing agents (e.g., hyaluronic acid).
Avoidable ingredients	Retinoids, arbutin, resorcinol derivatives, salicylic acid, and parabens.
Application frequency	Reapply as needed, especially after swimming, sweating, or prolonged sun exposure.
Special considerations	Prefer non-spray formulations to avoid inhalation risks.

*Effective protection against HEVL-induced pigmentation requires the presence of iron oxide in formulations.

#### 3.2.3 Endocrine disruptors: a critical concern during pregnancy

Endocrine disruptors are of particular concern during pregnancy, as this period is highly sensitive to hormonal disturbances. Filters such as oxybenzone, ethylhexyl methoxycinnamate (octinoxate) and 4-methylbenzylidene camphor have been associated with endocrine effects. To be prudent, the use of sunscreens containing these UV filters should be avoided during pregnancy. Regarding homosalate and octocrylene, the current body of evidence is insufficient to classify them as endocrine-disrupting substances (33–35).

In light of these considerations, all healthcare providers can recommend formulations prioritizing inorganic filters, potentially supplemented with safe organic filters with a good toxicological profile to optimize cosmetic acceptability and adherence. This balanced approach ensures effective photoprotection while addressing both safety and user experience.

#### 3.2.4 Non-filter active ingredients for sunscreen formulation

Sunscreens have become versatile products, particularly for pregnant and lactating women, where safety is paramount for both mother and child. Customization of sunscreen products include formulations that contain non-filter ingredients with anti-aging properties, such as hyaluronic acid and peptides. For those at risk of hyperpigmentation, safe agents are niacinamide and vitamin E (36). Azelaic acid with glycine and hesperidin-chalcone could be beneficial for pregnant women with facial erythema. It is crucial to avoid sunscreens that contain ingredients such as retinoids, arbutin, resorcinol derivatives, salicylic acid, and potential endocrine disruptors, to ensure safety during this sensitive period (37).

#### 3.2.5 Cosmetic elegance as a key factor for patient adherence

The sensory qualities of a sunscreen are essential for ensuring adherence and regular application. Key characteristics include a non-sticky texture, absence of residue, and a velvety, matte finish that provides a uniform complexion by minimizing visible pores and imperfections (38). Achieving these qualities requires extensive research and development by the sunscreen industry, including optimizing the blend of chemical (organic) and physical (inorganic) filters—sometimes utilizing nanotechnology to prevent the white cast from non-nanonized inorganic filters. These formulations are further enhanced with effective vehicles and other ingredients like hyaluronic acid for hydration and antioxidants, contributing to both skin protection and overall aesthetic appeal.

Concerns exist regarding the potential health impacts of nanoparticles (39–41). However, studies on the use of nanoparticles, especially zinc oxide (ZnO) and titanium dioxide (TiO2), in sunscreens have generally found them safe for use in the general population, including pregnant women. These nanoparticles are largely designed to remain on the skin’s outermost layer, the stratum corneum, without penetrating to viable cells. Research by regulatory bodies like the European Commission’s Scientific Committee on Consumer Safety (SCCS) and the Australian Therapeutic Goods Administration (TGA) supports that nanoparticles used in sunscreens do not reach systemic circulation in significant amounts, minimizing risks of adverse effects. In fact, among UV filters listed in the US FDA monograph, only titanium dioxide and zinc oxide are classified as “generally recognized as safe and effective (GRASE)” (42). However, spray formulations containing inorganic filters during pregnancy should be avoided when possible due to the risk of inhalation and absorption via the respiratory tract. This property, along with the protective benefits against UV exposure, suggests that the benefits outweigh potential risks for sensitive populations, including pregnant women (43).

## 4 Conclusion

Managing skin health during pregnancy requires careful and consistent photoprotection, with special consideration for the safety of both the pregnant individual and the developing baby. Data indicates that the advantages of routine UV filter application substantially exceed the minimal risks reported. Proactive use of broad-spectrum sunscreens can help prevent pregnancy-associated hyperpigmentation and reduce the risk of UV-induced skin damage. Given the heightened sensitivity to UV radiation and visible light during pregnancy, broad-spectrum sunscreens that protect against UVA, UVB, and high-energy visible light (HEVL) are essential. Mineral-based, tinted sunscreens containing iron oxides are particularly suitable for pregnant individuals of skin of color, as they provide both HEVL protection and cosmetic camouflage, and are generally considered safe during pregnancy and breastfeeding. Sunscreens combining mineral protection and safe organic filters can also be considered as they optimize cosmetic acceptability and adherence.

For individuals with skin tones that do not align with available tinted options, layering a non-tinted, high-protection sunscreen with makeup containing iron oxides is a practical alternative. Importantly, selecting sunscreens free from potentially harmful chemical filters, such as oxybenzone, can ensure additional safety for both mother and baby during pregnancy and breastfeeding.

Recent advances in understanding the impact of oxidative stress from UVA and VL exposure suggest that incorporating antioxidants into sunscreens may enhance photoprotection. By following these photoprotection guidelines, pregnant and breastfeeding individuals can mitigate pigmentation changes, protect their skin, and maintain safety for both themselves and their baby.
